# Changes in organ donations after the implementation of a controlled cardiac death (maastricht Type III) donation protocol

**DOI:** 10.1186/2197-425X-3-S1-A896

**Published:** 2015-10-01

**Authors:** S Alcantara Carmona, N Martínez Sanz, B Lobo Valbuena, J Palamidessi Domínguez, R Fernández Rivas, M Pérez Redondo, M Valdivia de la Fuente, B Balandín Moreno, JJ Rubio Muñoz

**Affiliations:** Intensive Care Unit, Hospital Universitario Puerta de Hierro Majadahonda, Majadahonda, Spain

## Objective

**Figure 1:**
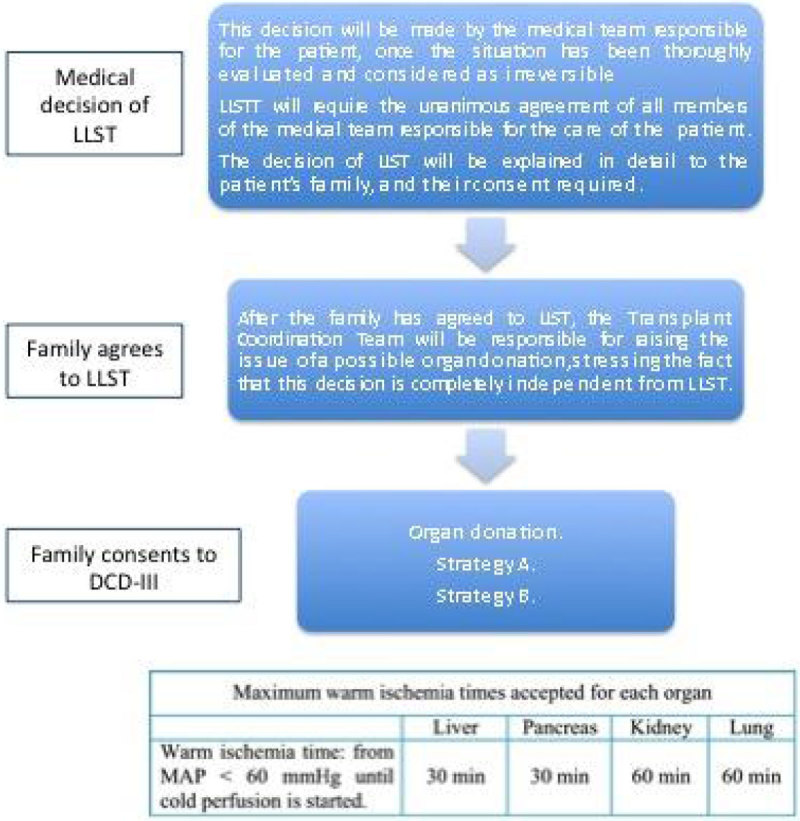
Figure 1

Due to the decrease in brain death donors (BDD), donation after controlled cardiac death (Maastricht III type patients, DCD-III) has aroused as another strategy of donation. The objective of this research is to study the general characteristics and the impact on donation rates of a DCD-III protocol (Figure 1), after its implementation (January 2012), in a tertiary hospital.

## Methods

**Figure 2:**
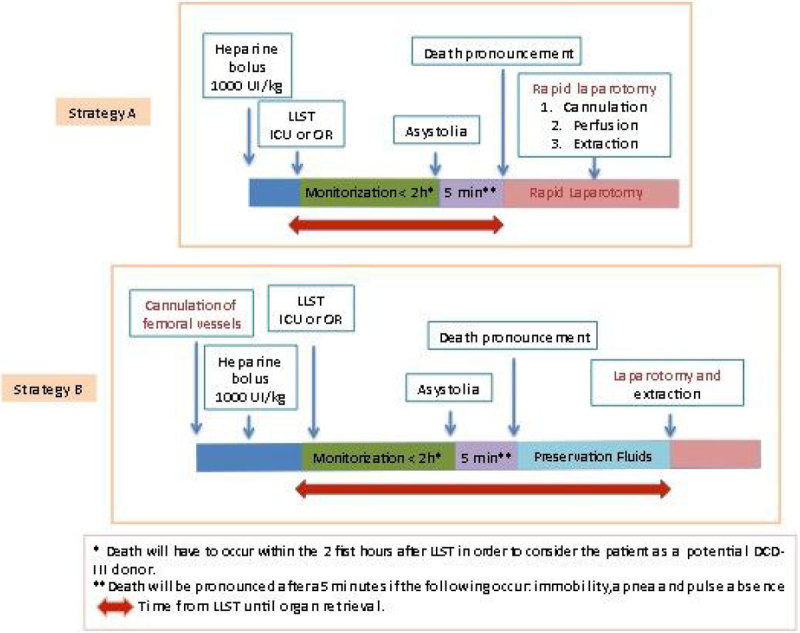
Figure 2

Retrospective, descriptive and observational study (January 2012-February 2015). The DCD-III protocol enclosed limitation of life sustaining therapies (LLST) in both the intensive care unit (UCI) and the operating room (OR), and included two strategies (Strategy A: rapid surgery and Strategy B: cannulation, Figure 2). Type and number of organs obtained, and their impact on donation rates were analyzed.

## Results

**Figure 3:**
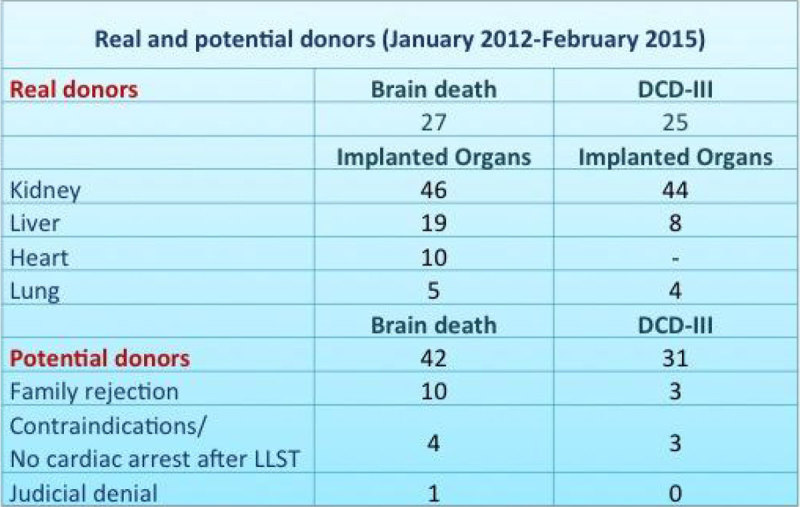
Figure 3

During the study period there were 73 potential donors that turned out in 52 real donors. BDD accounted for 27 donations and DCD-III for 25 (48% increase in donations after the introduction of DCD-III). In the DCD-III group, LLST was done in all patients but one in the OR. Eighteen patients underwent rapid surgery. Characteristics of donation and differences in organ retrieval are shown in Figure 3. In the 2010-2012 period, monthly organ donation rate was 0,45, and it tripled to 1,4 donors/month after the establishment of the DCD-III protocol. Mean organ rate recovered by donor was 2,96 for BDD and 2,24 for DCD-III. DCD-III donors were responsible for a 48,8%, 29,6% and 44,4% of the kidney, liver and lung transplants in the study period.

## Conclusions

DCD-III is a valid method for increasing organ donation rates. DCD-III programs should form part of organ donation strategies.

